# Retraction Note: LncRNA LIFR-AS1 promotes proliferation and invasion of gastric cancer cell via miR-29a-3p/COL1A2 axis

**DOI:** 10.1186/s12935-026-04367-3

**Published:** 2026-06-10

**Authors:** Haiyan Pan, Yuanlin Ding, Yugang Jiang, Xingjie Wang, Jiawei Rao, Xingshan Zhang, Haibing Yu, Qinghua Hou, Tao Li

**Affiliations:** 1 School of Public Health, Guangdong Medical University, Dongguan, 523808 Guangdong People’s Republic of China; 2 Department of gastrointestinal Surgery, Shandong Provincial Hospital, Jinan, 250021 Shandong People’s Republic of China; 3 College of Ocean and Meteorology, Guangdong Ocean University, Zhanjiang, 524088 Guangdong People’s Republic of China; 4 Department of Chemotherapy, The People’s Hospital of Gaozhou, Gaozhou, 525200 Guangdong People’s Republic of China

**Retraction Note: Cancer Cell International (2020) 21:7**

10.1186/s12935-020-01644-7

The Editors-in-Chief have retracted this article. Following the publication of this article, concerns were raised regarding Western blots and flow cytometry data presented in Figs. 1, 2 and 4. The authors were unable to provide raw data for further validation upon request.

The Editors-in-Chief therefore have lost confidence in the results and conclusions of this article.

None of the authors has responded to any correspondence regarding this retraction notice.

